# High Fat Diet Triggers a Reduction in Body Fat Mass in Female Mice Deficient for Utx demethylase

**DOI:** 10.1038/s41598-019-46445-9

**Published:** 2019-07-11

**Authors:** Kazushige Ota, Akiyoshi Komuro, Hisayuki Amano, Akinori Kanai, Kai Ge, Takeshi Ueda, Hitoshi Okada

**Affiliations:** 1 Department of Biochemistry, Kindai University Faculty of Medicine, Osaka-Sayama, Osaka Japan; 2 Department of Molecular Oncology, Research Institute for Radiation Biology and Medicine, Hiroshima University, Hiroshima, Japan; 3 Adipocyte Biology and Gene Regulation Section, Laboratory of Endocrinology and Receptor Biology, National Institute of Diabetes and Digestive and Kidney Diseases, National Institutes of Health, Bethesda, MD 20892 USA

**Keywords:** Epigenetics, Endocrine system and metabolic diseases

## Abstract

Obesity increases the risk of metabolic disorders like diabetes mellitus and dyslipidemia. However, how metabolic status is sensed and regulates cellular behavior is unclear. Utx is an H3K27 demethylase that influences adipocyte function *in vitro*. To examine its role *in vivo*, we generated mice lacking Utx in adipocytes (UtxAKO). Although all UtxAKO mice grew normally on a normal chow diet (NCD), female UtxAKO mice on a high fat diet (HFD) showed striking reductions in body fat compared to control mice (Ctrl). Gene expression profiling of adipose tissues of HFD-fed UtxAKO female mice revealed decreased expression of rate-limiting enzymes of triacylglycerol synthesis but increased expression of those of cholesterol/steroid hormone synthesis. Moreover, these animals resisted adiposity induced by ovariectomy and exhibited increased estrogen in visceral adipose tissues. Thus, upon HFD feeding, Utx regulates lipid metabolism in adipose tissues by influencing the local hormonal microenvironment. Conversely, Utx deficiency skews lipid catabolism to enhance cholesterol/steroid hormone production and repress obesity.

## Introduction

Obesity is a complex disease caused by a prolonged imbalance between energy intake and expenditure. Extensive genetic studies have identified obesity-related loci as being functionally linked to hypothalamic regulators of appetite and glucose, lipid or energy metabolism, hormone and nuclear hormone binding, and immune processes^1–5^. However, the control of lipid metabolism is highly dynamic and depends on metabolic demands and supplies. White adipose tissue (WAT) is a key regulator of energy balance and lipid metabolism because it stores excessive energy as triglyceride (TG). When the stored TG in WAT is mobilized, free fatty acids (FA) are released due to the actions of lipases, adipose triglyceride lipase (ATGL), hormone-sensitive lipase (HSL) and monoacylglycerol lipase (MGL). The importance of these enzymes in lipid homeostasis is highlighted by the abnormal fat accumulation and adipocyte hypertrophy observed in ATGL-deficient and HSL-deficient mice^6,7^. Significantly, WAT also functions as an endocrine organ and secretes adipokines that control lipid metabolism, inflammation and insulin sensitivity^8^.

Sterol regulatory element-binding proteins (SREBPs) are a family of transcription factors that regulate multiple enzymes required for the biosynthesis of cholesterol and FA^9^. SREBPs contain three isoforms, SREBP-1a, -1c and 2. The SREBP-1 isoforms are more selective in activating genes involved in FA synthesis, while SREBP-2 is more specific for genes involved in cholesterol synthesis^10^. Steroid hormones, affecting lipid and energy homeostasis, are synthesized from cholesterol. Among these hormones, estrogen plays a key part in regulating lipid metabolism. Postmenopausal women tend to gain body weight and show increased adiposity^11,12^, whereas postmenopausal women on hormone replacement therapy show a decrease in age-related weight gain^13,14^. In addition to the systemic estrogen produced by the ovaries, peripheral estrogen is synthesized within adipocytes via aromatization of androgenic precursors by aromatase^15^. The importance of this peripheral estrogen production in regulating adiposity and lipid metabolism is underscored by the phenotypes of aromatase-deficient mice, which exhibit increased adiposity, insulin resistance and glucose intolerance^16–18^.

Utx (also called Kdm6a) is a histone H3K27me2/3 demethylase that is essential for endoderm differentiation and cardiac and neural tube development in mice^19,20^. The *Utx* gene is located on the X chromosome in both humans and mice. In humans, *UTX* mutations have been identified in a subset of patients with Kabuki syndrome (KS), a rare pediatric congenital disorder characterized by facial and skeletal abnormalities^21,22^ with occasional endocrinological symptoms^23–28^; however, the underlying etiology is currently unknown. *In vitro*, Utx deficiency impaired adipogenesis in mouse embryonic stem cells^29^. Thus, Utx appears to control adipose tissue homeostasis and lipid metabolism and therefore may be influential in obesity, but the molecular mechanisms involved are still unclear, especially *in vivo*. We explored this issue using adipocyte-specific Utx-deficient mice.

## Results

### Female UtxAKO mice are protected against obesity induced by HFD

To better understand the role of Utx in adipose tissues and lipid metabolism, we crossed mice carrying a floxed Utx allele^30^ with mice carrying the aP2 promoter-Cre transgene^31^ to generate progeny lacking Utx in adipocytes as well as the appropriate control littermates. Control Utx^flox/flox^ (Ctrl) and Utx^flox/flox^;aP2-Cre (UtxAKO) mice were viable, and had no macroscopic abnormalities (data not shown). The deletion of the floxed Utx allele was observed in the WAT and brown adipose tissue (BAT) of UtxAKO mice but not in Ctrl littermates (Supplementary Fig. 1A). The appearance of incomplete Utx deletion in WAT is due to its composition of several different cell types in addition to adipocytes, including fibroblasts and blood cells; indeed, 30% of cells in adipose tissue are non-fat cells^32^. At the protein level, Utx was reduced in WAT and BAT of UtxAKO mice but not in liver (Supplementary Fig. 1B), confirming the Utx deletion in the adipocytes of UtxAKO mice.

**Figure 1:**
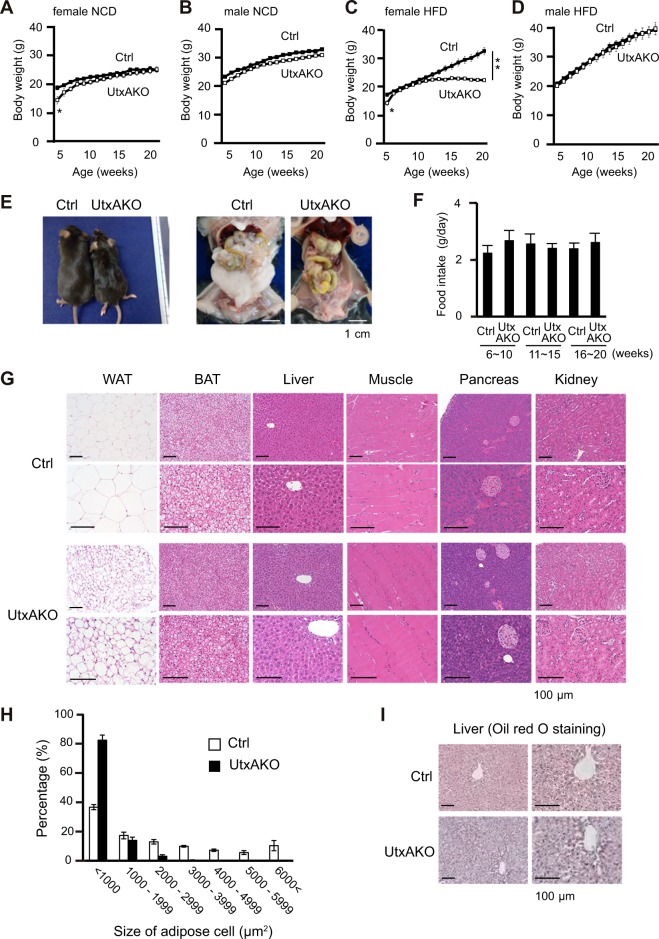
Female UtxAKO mice fed on a HFD show reduced adiposity. (**A**–**D**) Quantitation of change in body weight (BW) with time: (**A**) f-NCD-Ctrl vs. f-NCD-UtxAKO mice (*n* = 5/group); (**B**) m-NCD-Ctrl vs. m-NCD-UtxAKO mice (Ctrl *n* = 4, UtxAKO *n* = 6); (**C**) f-HFD-Ctrl vs. f-HFD-UtxAKO mice (Ctrl *n* = 10, UtxAKO *n* = 8); (**D**) m-HFD-Ctrl vs. m-HFD-UtxAKO mice (*n* = 5/group). (**E**) Left panel: Representative gross appearance of f-HFD-UtxAKO (right) and f-HFD-Ctrl (left) mice at 20 weeks of age. Middle panel: Increased visceral WAT (compared to f-NCD-Ctrl) in a f-HFD-Ctrl mouse at 20 weeks. Right panel: Decreased visceral WAT (compared to f-HFD-Ctrl) in a f-HFD-UtxAKO mouse at 20 weeks. Magnifications are shown (scale bars, 1 cm). (**F**) Quantitation of food intake by f-HFD-Ctrl vs. f-HFD-UtxAKO mice (*n* = 7/group) at the indicated ages. (**G**) H&E staining of the indicated tissues of f-HFD-Ctrl vs. f-HFD-UtxAKO mice at 20 weeks. Two magnifications are shown (scale bars, 100 μm). (**H**) Quantification of the lipid droplets with the indicated area size in visceral WAT of f-HFD-Ctrl vs. f-HFD-UtxAKO mice at 20 weeks (mean ± SD; *n* = 5/group). (**I**) Representative images of Oil red O staining of livers of f-HFD-Ctrl and f-HFD-UtxAKO mice at 20 weeks (*n* = 5/group). Two magnifications are shown (scale bars, 100 μm). For all Figures, data are the mean ± SEM and are representative of at least three independent trials. *p < 0.05, **p < 0.005.

To assess the effect of adipocyte-specific Utx deficiency on lipid metabolism, male and female Ctrl and UtxAKO mice were fed on either a normal chow diet (NCD) or a high fat diet (HFD) (Supplementary Fig. 1C). We first monitored body weight (BW) of these animals over time. The BW of both female and male UtxAKO mice on NCD overtime was similar to that of Ctrl mice (Fig. 1A,B). Unexpectedly we found that female HFD-fed UtxAKO mice (f-HFD-UtxAKO) gained much less weight than their female HFD-fed Ctrl (f-HFD-Ctrl) counterparts (Fig. 1C). The decrease in BW was common across most of f-HFD-UtxAKO mice (Supplementary Table 1). This difference was not apparent in comparisons of male HFD-Ctrl and HFD-UtxAKO mice (Fig. 1D). The profound reduction in body size of f-HFD-UtxAKO mice compared to f-HFD-Ctrl mice was clear upon gross examination at 20 weeks of age, as was the decrease in the volumes of their visceral and subcutaneous adipose tissues upon dissection (Fig. 1E). Food intake over 6–20 weeks was comparable between f-HFD-UtxAKO and f-HFD-Ctrl mice (Fig. 1F), ruling out a decrease in overall nutrition as the cause of the BW reduction in f-HFD-UtxAKO mice. In addition, the BW of f-UtxAKO and f-Ctrl mice overtime was equal when fed on a high glucose diet (HGD) (Supplementary Fig. 1D), implying that the BW reduction in f-HFD-UtxAKO mice was specifically related to the increased fat content of the HFD.

To gain further insights into the lean phenotype of f-HFD-UtxAKO mice, we performed histological analyses of various tissues at age 20 weeks. The visceral WAT of f-HFD-UtxAKO mice contained smaller adipocytes with less stored lipid, whereas the BAT, muscle, pancreas, and kidney showed no differences between f-HFD-UtxAKO and f-HFD-Ctrl mice (Fig. 1G). The percentage of smaller lipid droplets in visceral WAT was higher in f-HFD-UtxAKO mice than f-HFD-Ctrl mice (Fig. 1H). Oil red O staining revealed that the lipid content of hepatocytes in f-HFD-UtxAKO mice was decreased compared to f-HFD-Ctrl mice (Fig. 1I). In contrast, all tissues of f-NCD-UtxAKO mice, including BAT and visceral WAT, were similar in morphology to those of f-NCD-Ctrl mice (Supplementary Fig. 2A). These results indicate that HFD triggered a reduction in BW and body fat mass in female UtxAKO mice that did not occur in HFD- or NCD-fed UtxAKO male mice or in HGD- or NCD-fed UtxAKO female mice. Since this effect was observed only in female UtxAKO mice, we used females for all subsequent experiments.

### Female HFD-UtxAKO mice exhibit increased serum lipid levels and attenuated hepatic steatosis

**Figure 2:**
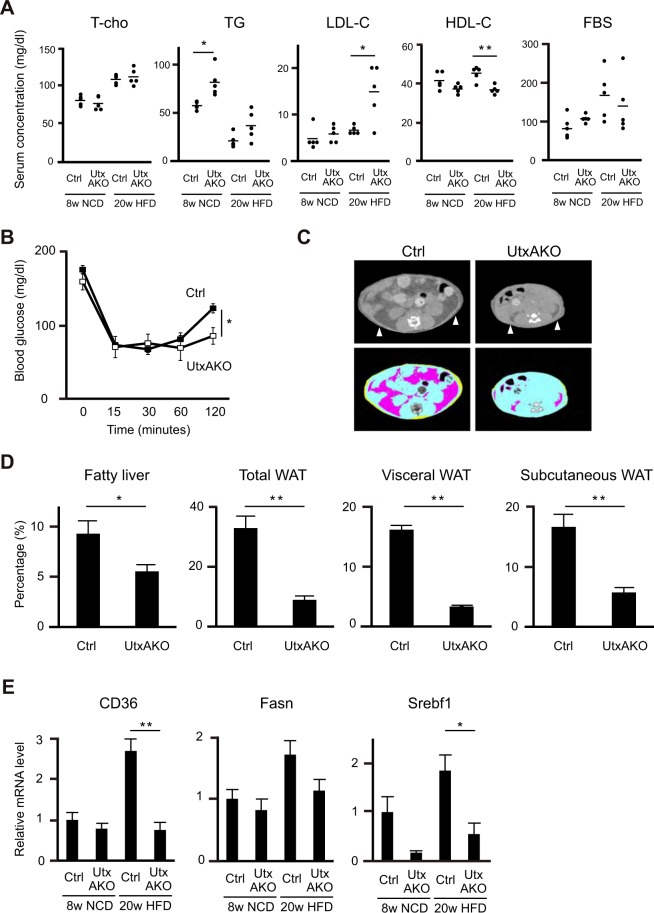
Metabolic profiling of female UtxAKO mice on HFD. (**A**) Quantitation of serum levels of the indicated metabolites in f-NCD-Ctrl and f-NCD-UtxAKO mice at 8 weeks of age, and in f-HFD-Ctrl and f-HFD-UtxAKO mice at 20 weeks of age (*n* = 5/group). T-cho, total cholesterol; TG, triacylglycerol; LDL-C, LDL cholesterol; HDL-C, HDL cholesterol; FBS, fasting blood glucose after 16 hr starvation. (**B**) Time course of blood glucose levels in f-HFD-Ctrl and f-HFD-UtxAKO mice (*n* = 5/group) that were fasted for 3 hr and received i.p. injection of insulin (0.75 mIU/g BW). Plasma glucose levels were measured at the indicated time points. (**C**) Representative CT images of abdomens of f-HFD-Ctrl and f-HFD-UtxAKO mice at 20 weeks (*n* = 7/group). CT images at the same sectional levels were compared. Upper panels: Arrowheads indicate low density areas corresponding to fat. Lower panels: Pink and yellow areas indicate visceral and subcutaneous fats, respectively. (**D**) Quantitation of hepatic steatosis (fatty liver) and percentage of total WAT, visceral WAT and subcutaneous WAT in f-HFD-Ctrl and f-HFD-UtxAKO mice at 20 weeks (*n* = 7/group). (**E**) Quantitative RT-PCR determination of mRNA levels of the indicated genes in livers of f-NCD-Ctrl and f-NCD-UtxAKO mice at 8 weeks, and f-HFD-Ctrl and f-HFD-UtxAKO mice at 20 weeks (*n* = 5/group). Results are expressed relative to the mRNA level of TBP (internal control).

Further examination of metabolic parameters revealed that serum TG levels were higher in f-NCD-UtxAKO mice than in f-NCD-Ctrl animals at 8 weeks of age, but that total cholesterol (T-cho), LDL cholesterol (LDL-C), HDL cholesterol (HDL-C) and fasting blood glucose (FBS) levels were comparable (Fig. 2A). In contrast, after 15 weeks on HFD (20 weeks of age), f-HFD-UtxAKO mice exhibited higher TG and LDL-C but lower HDL-C than f-HFD-Ctrl mice (Fig. 2A). T-cho levels in f-HFD-UtxAKO mice were similar to those in f-HFD-Ctrl mice, but fasting blood glucose (FBS) levels tended to be lower in f-HFD-UtxAKO mice (Fig. 2A). Consistent with this finding, f-HFD-UtxAKO mice showed the prolonged response to insulin, in that insulin administration decreased glucose levels to below those in similarly treated f-HFD-Ctrl mice (Fig. 2B). Parallel tests of glucose or insulin tolerance conducted in NCD-fed mice showed no differences (Supplementary Fig. 2B,C). Thus, loss of Utx allows female UtxAKO mice to maintain higher serum lipid levels and the prolonged response to insulin in the presence of the elevated fat of HFD.

We confirmed the above results by measuring body fat mass using computed tomography (CT). A comparison of cross-sectional images of the abdomens of f-HFD-UtxAKO and f-HFD-Ctrl mice at same level indicated that the mutants exhibited a reduction in fat mass (Fig. 2C). Quantification with CT revealed that the adiposity and volume of the visceral and subcutaneous WAT were decreased in f-HFD-UtxAKO mice compared to f-HFD-Ctrl mice, and that the fat content in the liver of f-HFD-UtxAKO mice was also decreased (Fig. 2D and Supplementary Fig. 3A). The body length of f-HFD-UtxAKO mice was similar to that of f-HFD-Ctrl mice, showing that Utx deficiency in adipose tissue did not affect the body length at 20 weeks of age on HFD (Supplementary Fig. 3B). Lastly, we analyzed expression levels of genes associated with lipid metabolism and hepatic steatosis in the livers. The mRNA levels of genes responsible for FA uptake (*CD36*) and lipogenesis (*Fasn*, *Srebf1*) were reduced in f-HFD-UtxAKO liver (Fig. 2E), consistent with its decreased lipid content. Thus, despite slightly increasing serum lipid levels, Utx deficiency in adipose tissues protects against the adiposity, hepatic steatosis induced by consumption of HFD.

### Utx controls the expression of genes involved in cholesterol and estrogen synthesis

**Figure 3:**
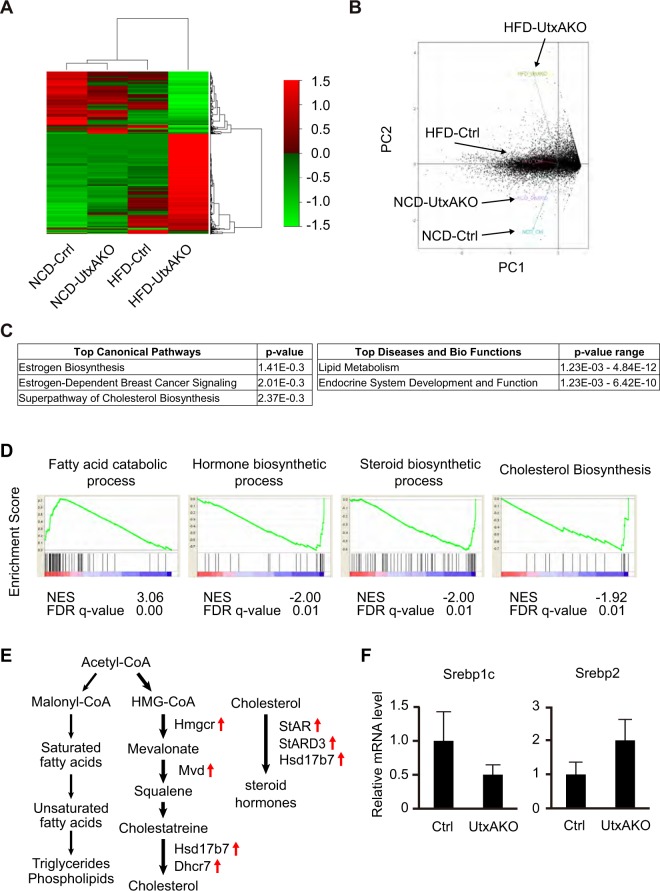
Utx deficiency represses triglyceride synthesis and induces cholesterol and estrogen synthesis. (**A**) Unsupervised clustering of gene expression patterns in visceral WAT of f-NCD-Ctrl, f-NCD-UtxKO, f-HFD-Ctrl and f-HFD-UtxAKO mice at 13 weeks, as indicated (n = 3). Right side: Corresponding heat map of barcode representation. Red and green correspond to high and low expression levels, respectively, compared with the experiment-wide median. The averages of each group with three mice were used for the clustering. (**B**) Principal component analysis (PCA) based on the gene expression profiles in visceral WAT of the mice in (**A**). (**C**) Identification of estrogen synthesis, estrogen signaling pathways and lipid metabolism among five top hits of Top Canonical Pathways and Top Diseases and Bio Functions in the f-HFD-UtxAKO mice by Ingenuity pathway analysis (IPA). The differentially expressed genes between f-HFD-Ctrl and f-HFD-UtxAKO with statistical significance were analyzed with IPA. Results are shown with *p*-values. (**D**) Identification of enhanced cholesterol/estrogen biosynthesis and decreased fatty acid synthesis in HFD-fed UtxAKO mice compared to HFD-fed Ctrl by Gene Set Enrichment Analysis (GSEA) at 13 weeks. The analysis examined isoforms that were expressed more than 0.3 FPKM (Fragments Per Kilobase of exon per Million mapped reads) in visceral WAT of f-HFD-Ctrl vs. f-HFD-UtxAKO mice (n = 3). Results associated with cholesterol/estrogen metabolism are shown among the gene sets with FDR *q*-value < 0.25. (**E**) Schematics of *de novo* lipogenesis and estrogen metabolism. The expression levels of the indicated rate limiting enzymes for cholesterol/estrogen syntheses were upregulated in the f-HFD-UtxAKO mice. (**F**) Quantitative RT-PCR analyses of mRNA levels of the indicated genes in visceral WAT of f-HFD-Ctrl and f-HFD-UtxAKO mice at 13 weeks (*n* = 5/group). Results are expressed relative to the mRNA level of TBP (internal control).

To gain mechanistic insights into the lean phenotype of f-HFD-UtxAKO mice, we performed gene expression profiling of the visceral WAT of NCD- or HFD-fed Ctrl and UtxAKO female mice at 13 weeks of age, the time when the deceleration in mutant BW gain just becomes obvious. First, we clustered the gene expression profiles of f-NCD-Ctrl, f-NCD-UtxAKO, f-HFD-Ctrl and f-HFD-UtxAKO mice (Fig. 3A). The averages of three mice from each set were used for analysis. This exercise showed that the gene expression profiles of f-NCD-Ctrl, f-NCD-UtxAKO, and f-HFD-Ctrl mice were generally comparable, whereas the profile of f-HFD-UtxAKO mice contained differentially expressed gene clusters (Fig. 3A). The clustering between separate three f-HFD-Ctrl and f-HFD-UtxAKO mice were shown in Supplementary Fig. 4. A subsequent Principal Component Analysis (PCA) confirmed this dramatic difference (Fig. 3B). We next performed an Ingenuity Pathway Analysis (IPA) to identify pathways that were associated with these component/gene expression profiles and were enriched in adipose tissues of f-HFD-UtxAKO mice. The Top Canonical Pathways identified were linked to estrogen metabolism, estrogen-dependent breast cancer signaling, and superpathway of cholesterol biosynthesis (Fig. 3C). The IPA also identified the Top Diseases and Bio Functions associated with the gene expression pattern in f-HFD-UtxAKO adipose tissues to be linked to lipid metabolism and endocrine system development and function (Fig. 3C). These data bolster and extend our CT and qRT-PCR results.

To delve deeper into the differentially expressed gene profiles identified above, we performed a Gene Set Enrichment Analysis (GSEA) comparing f-HFD-Ctrl and f-HFD-UtxAKO mice. While genes associated with FA catabolism were upregulated in f-HFD-Ctrl adipose tissue, the expression levels of genes linked to hormone biosynthesis, steroid biosynthesis, and cholesterol biosynthesis were enriched in f-HFD-UtxAKO adipose tissue (Fig. 3D). We therefore hypothesized that, in animals where FA synthesis was suppressed by HFD consumption, Utx deficiency might facilitate *de novo* estrogen synthesis and activate estrogen signaling in adipose tissue. To test this theory, we compared mRNA levels of the rate-limiting enzymes in FA synthesis and cholesterol/steroid hormone synthesis in adipose tissues of f-HFD-UtxAKO mice versus f-HFD-Ctrl mice. In mutant adipose tissue, we found upregulation of the genes responsible for cholesterol synthesis (*Hmgcr*, *Mvd*, *Hsd17b7*, and *Dhcr7*) and steroid hormone synthesis (*StAR*, *StARD3*, and *Hsd17b7*), but downregulation of the genes driving TG synthesis from the lists of GSEA (Fig. 3E, Supplementary Fig. 5). Next we quantified the expression levels of SREBPs, critical regulators of lipid biosynthesis. SREBP-1c is more selective for inducing the genes for FA/TG synthesis, while SREBP-2 is more specific for inducing the genes for cholesterol synthesis. In supporting the upregulation of the genes responsible for cholesterol/steroid hormone synthesis and downregulation of the genes driving TG synthesis in UtxAKO adipose tissues, *Srebp1c* expression levels were down regulated and *Srebp2* were upregulated in UtxAKO mice (Fig. 3F). The expression levels of *Srebp1a* were similar between Ctrl and UtxAKO mice (Supplementary Fig. 6). We confirmed the upregulation of the genes involved in cholesterol/steroid hormone synthesis in several Ctrl and UtxAKO mice (Supplementary Fig. 6). These data prompted us to consider a model in which Utx deficiency alters lipid metabolism by skewing the balance among the TG, cholesterol and steroid hormone synthetic pathways in response to HFD feeding.

### Upregulated estrogen production prevents obesity induced by HFD and ovariectomy in female UtxAKO mice

**Figure 4:**
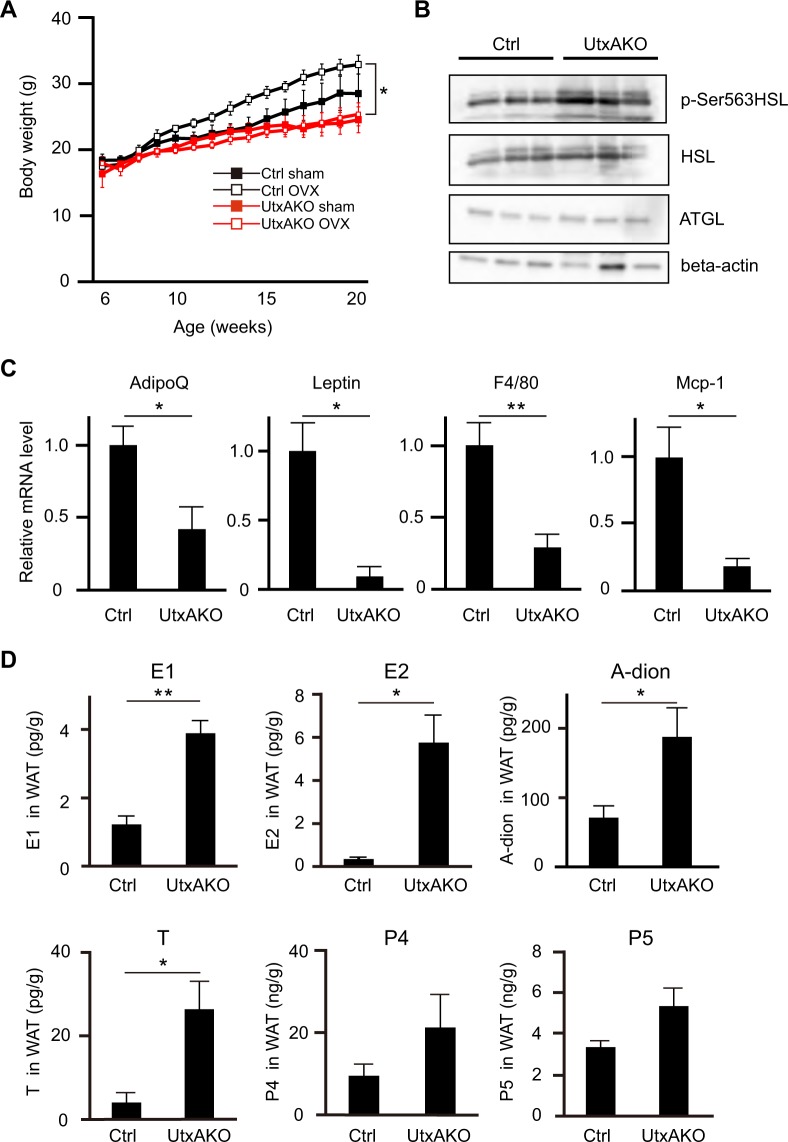
Female UtxAKO mice resist obesity induced by ovariectomy (OVX) and HFD. (**A**) Time course of BW gain in f-HFD-Ctrl and f-HFD-UtxAKO mice that were subjected to sham surgery or OVX at 6 weeks of age (*n* = 4/group) and fed on HFD until 20 weeks of age. (**B**) Protein levels of the lipases were examined in visceral WAT of HFD-fed female Ctrl and UtxAKO mice at 20 weeks of age (*n* = 3). HSL (hormone-sensitive lipase), phosphor-HSL (Ser563), ATGL (adipose triglyceride lipase), beta-actin. (**C**) Relative mRNA levels of the indicated genes in visceral WAT of f-HFD-Ctrl and f-HFD-UtxAKO mice at 20 weeks of age (*n* = 5/group). (**D**) Increased concentrations of E1, E2, A-dion, T, P4, and P5 in visceral WAT of HFD-fed UtxAKO female mice. The concentration of E1, E2, A-dion, T, P4, and P5 in visceral WAT were quantified by mass-spectrometry from HFD-fed Ctrl and UtxAKO mice with OVX at 20 weeks of age (*n* = 4). Values in graphs are expressed as mean ± SE. *p < 0.05, **p < 0.005.

We sought to test our theory in the absence of any confounding effects due to systemic estrogen production by the ovaries. To this end, we subjected Ctrl and UtxAKO female mice to ovariectomy (OVX) so as to highlight peripheral estrogen production. We then compared the BW gain of OVX-Ctrl and OVX-UtxAKO mice fed on HFD. We found that OVX-f-HFD-Ctrl mice gained more weight than f-HFD-Ctrl mice, as expected (Fig. 4A). However, the BWs of OVX-f-HFD-UtxAKO mice were comparable to those of f-HFD-UtxAKO mice, implying that it is the local production of estrogen that is critical for the BW loss phenotype of f-HFD-UtxAKO mice.

We next applied immunoblotting to the visceral WAT of f-HFD-UtxAKO and f-HFD-Ctrl mice to compare protein expression of elements of the main signaling pathways responsible for lipolysis. We found that HSL phosphorylation was increased in f-HFD-UtxAKO WAT compared to f-HFD-Ctrl WAT (Fig. 4B). This enhanced lipolysis could account for the increased serum levels of TG and LDL-C in these animals (as shown in Fig. 2A). Levels of ATGL were similar in f-HFD-UtxAKO WAT and f-HFD-Ctrl WAT. Because adipokines and inflammation also influence lipid metabolism in adipose tissue, we determined the mRNA levels of the adipokines adiponectin and leptin as well as the inflammatory markers *F4/80* and *Mcp-1* in f-HFD-UtxAKO WAT and f-HFD-Ctrl WAT. We found that all four of these molecules were decreased in f-HFD-UtxAKO WAT compared to f-HFD-Ctrl WAT (Fig. 4C), indicating that Utx deficiency reduces adipokine production and represses the inflammation invoked by HFD.

Lastly, to further establish *in vivo* the crucial function of steroid hormones in the f-HFD-UtxAKO phenotype, we used quantitative LC-MS/MS to measure levels of the hormones, estrone (E1), 17β-estradiol (E2), androstendion (A-dion), testosterone (T), progesterone (P4), and pregnenolone (P5) in WAT of OVX-f-HFD-Ctrl and OVX-f-HFD-UtxAKO mice. The concentrations of E1, E2, A-dion, and T in OVX-f-HFD-UtxAKO WAT were significantly higher than those in OVX-f-HFD-Ctrl WAT (Fig. 4D). These data confirm that peripheral estrogen synthesis is enhanced in HFD-fed UtxAKO female mice, and suggest that this upregulation of sex steroid hormones in adipose tissues can repress HFD-induced obesity.

## Discussion

In this study, we demonstrate that Utx deficiency in adipocytes prevents an increase in body fat mass in female mice fed on a HFD. RNA sequence analysis and quantitative mass spectrometry revealed enhanced steroid hormone synthesis in the visceral adipose tissues of f-HFD-UtxAKO mice that was not observed in f-HFD-Ctrl or f-NCD-UtxAKO animals. Thus, our work identifies a novel mechanism regulating the balance between peripheral steroid hormone production and FA/TG synthesis in response to excess fat intake. Interestingly, this increase in peripheral steroid hormone production in adipose tissue can prevent the adiposity induced by ovariectomy. Thus, our study has uncovered a new layer of regulatory control connecting nutritional environment to hormonal microenvironment and lipid metabolism.

Lipid homeostasis is maintained by controlling dietary intake, transport and lipid biosynthesis. A family of SREBPs plays a pivotal role in regulating the expression of lipogenic and cholesterogenic enzymes. SREBP-1c enhances FA/TG synthesis, while SREBP-2 accelerates cholesterol synthesis. We found that Srebp1c was downregulated, while *Srebp2* was upregulated in UtxAKO adipose tissues. These data suggest that Utx regulates the balance between the synthesis of cholesterol/steroid hormone and FA/TG at least in part by influencing the expression levels of *Srebps* in response to excess fat intake.

The mobilization of stored TG in WAT involves the release of free FA by lipases, ATGL, HSL and MGL^33^. Although controversies remain, it is widely accepted that estrogen enhances lipolysis. For example, free FA concentrations are decreased in OVX mice but rise after E2 administration^34^. Furthermore, visceral WAT is a major site of lipolysis impairment in OVX mice^35^, and the translocation of ATGL to lipid droplets blunts HSL phosphorylation in OVX mice^36^. One explanation for the increased HSL phosphorylation levels in our f-HFD-UtxAKO mice is estrogen’s potentiation of catecholamine- or glucagon-induced lipolysis. In support of this model, epinephrine- or glucagon-induced lipolysis is enhanced by estrogen treatment through activation of adenylate cyclase activity^37–39^.

We frequently observed lower BW in 4-week-old f-NCD-UtxAKO mice, the time when these pups are weaned from their mother. However, within two weeks of continued NCD feeding, the BW gain curve of these mutants became steeper and their BWs reached those of Ctrl littermates by 6 weeks of age. The fat content of mouse mother’s milk generally ranges from 13.5–21.4%^40–42^. Thus, this maternal “HFD” may have contributed to the initially lower BW of UtxAKO mice prior to their weaning. Although additional investigation is required to confirm this hypothesis, it may have significant implications for weight management in humans; that is, if the influence of Utx loss on lipid metabolism is reversible, it could offer new ways to control body weight, lipid metabolism, and the hormonal microenvironment merely by adjusting the fat content of food consumed.

The aP2-Cre mice we used express Cre in WAT, BAT, adipocyte precursors and CD31^+^ endothelial cells in adipose depots with much higher Cre-recombination in adipocytes of WAT (ranging from 50–80%) and negligible Cre-recombination in the liver and skeletal muscle^43^. The higher recombination efficiency in WAT is consistent with our data and the previous report^31^. However, our current model has not formally verified whether Utx-deficiency in mature adipocytes, adipocyte precursors or both contribute to the HFD-induced lean phenotype. In any case, further analyses using Adiponectin-Cre (specific for mature adipocytes in WAT)^44^ and PdgfRα-Cre (specific for adipocyte precursors)^45^ mice will clarify these issues.

*UTX* mutations occur in a subset of Kabuki syndrome (KS) patients^21,22^. About 25% of female KS patients exhibit premature thelarche^23–28^ but true central precocious puberty is rare^28,46,47^. Some cases of premature thelarche may involve the hypothalamus^28,47^ but the etiology of the remainder is unknown and thought to be heterogeneous. Based on our data, f-HFD-UtxAKO mice show increased estrogen synthesis in visceral adipose tissue. In addition, some of the genes involved in cholesterol/steroid hormone biosynthesis were upregulated in inguinal fat pad (data not shown). Thus, it is tempting to speculate that enhanced estrogen synthesis in mammary fat might contribute to the precocious puberty in some KS patients with UTX deficiency. Clearly, future clinical investigations are required to substantiate this theory.

Epigenetic control that appropriately shapes gene expression programs is critical for securing an effective response to a dynamically changing nutrient environment. Many studies have now established that proper histone methylation is vital for the regulation of energy expenditure, fat metabolism and adipogenesis *in vivo*^48^. We have provided compelling new evidence that Utx inactivation in adipocytes significantly changes local lipid metabolism and has systemic metabolic effects *in vivo*. Thus, our work significantly advances our understanding of the complexity of epigenetics-mediated metabolic regulation *in vivo*. Identification of ways to reversibly control the activity and timing of cholesterol/steroid hormone synthesis in adipose tissues could lead to an attractive therapeutic option for obesity and metabolic disorders in the future.

## Methods

### Animals and diets

The generation of Utx-floxed (Utx^flox/flox^) mice has been described previously^30^. B6.Cb-Tg(Fabp4-cre)1Rev/J (stock no. 005069) mice were obtained from the Jackson Laboratory. Fabp4-Cre, also known as aP2-Cre, transgene has a mouse *Fabp4/aP2* promoter directing Cre recombinase expression in brown and white adiopose tissue^31^. The Fabp4/aP2-Cre mice have been extensively used for adipose tissue functions^31,49,50^. All mice were bred and maintained in our facility and used their offspring in this study under germ-free conditions with a 12 hr light/dark cycle at a steady temperature of 22 °C at the Kindai University Faculty of Medicine (KUFM) animal facility. Mice were weaned at 4 weeks of age. A pair of Ctrl and UtxAKO mice from the same litter was housed in the same cage. Both male and female mice were studied. The composition of diet nutrition is in Supplementary Table 2. The measurement of body weight and body length, and the examination of morphology (macroscopic, tissue), serum biochemical parameters (total cholesterol, triacylglycerol, LDL-C, HDL-C, glucose), CT scan (adiposity, adipose volume, hepatic steatosis), tolerance test (glucose, insulin) were performed. For phenotypic characterization studies, age- and sex-matched mice were used. Mice were routinely maintained on a normal chow diet (NCD) containing 11.9% of calories from fat (CE-2, CLEA Japan). Beginning at 5 weeks of age, some mice were fed a high fat diet (HFD) containing 56.7% of calories from fat (HFD32, CLEA Japan) or high glucose diet (HGD) containing 50% of granulated sugar (AIN76, CLEA Japan) to 20 weeks of age. In some experiments, ovariectomy was performed on anesthetized mice at 6 weeks of age according to standard protocols. Food intake was measured from individual mice housed in isolated cages. We have chosen 8 weeks, 13 weeks and 20 weeks of age for the analyses because of the following reasons: (1) UtxAKO mice are smaller in size when they are weaned; however, they reached the same BW with Ctrl at 8 weeks of age. Thus, we used them for phenotypic analysis; (2) we used HFD-fed Ctrl and UtxAKO female mice at 13 weeks of age, the time when the deceleration in HFD-fed UtxAKO BW gain just becomes obvious, for gene expression analysis. We reasoned that the causal gene expression patterns could be identified from them, which may be masked at later time point because of systemic metabolic changes observed in the mice at 20 weeks of age; (3) BW loss in UtxAKO was much more obvious at 20 weeks of age. Thus, we used them for morphological analyses.

### Ethics statement

This study was conducted in accordance with Japanese animal protection law, all applicable federal and institutional regulations, and the policies and guidelines of the Japanese Council on Animal Care and the Japanese Association of Laboratory Animal Facilities of National University Corporations. All experiments were approved by the Local Institutional Animal Care and Research Advisory Committee of Kindai University Faculty of Medicine (KUFM). The KUFM animal facilities are committed to the highest ethical standards of care for animals used for the purpose of continued progress in the field of human medicine.

### Metabolic analyses

Mice were fasted for 16 hr (18:00 to 10:00) and euthanized with carbon dioxide at the indicated ages (8, 13, 20 weeks of age). The left cardiac ventricle was punctured and blood samples were collected in tubes with serum separating agent (Capijet, Terumo). For glucose tolerance tests, mice were fasted for 16 hr (18:00 to 10:00) before receiving intraperitoneal (i.p.) injection of 2 mg/g BW of glucose. Plasma glucose levels in tail vein blood samples were measured using a glucometer (Glucocard Arkray). For insulin tolerance tests, mice were fasted for 3 hr (10:00 to 13:00) prior to receiving i.p. injection of 0.75 mIU/g BW of insulin (Eli Lilly). Plasma glucose was determined as above. The both tolerance tests were conducted according to the previous reports^51,52^. Mice were allowed to recover between the glucose and insulin tolerance test for seven days.

### Computed tomography

Mice at 20 weeks of age were anesthetized and monitored throughout for general well-being. CT data capture and image reconstitution were performed using a Latheta LCT-200 scanner (Hitachi Aloka). Body fat and liver were analyzed quantitatively using Latheta software (ver 3.61) according to the manufacture’s instruction.

### Histology

Tissue samples were fixed with 10% formaldehyde. Paraffin-embedded sections were sectioned at 5 μm thickness and analyzed after hematoxylin and eosin staining. For Oil red O staining, after fixed with 10% formaldehyde, samples were incubated with 10% sucrose overnight followed by 20% sucrose overnight, frozen in OCT compound (Tissue-Tek). Samples were washed with isopropanol and stained with Oil red O (Sigma-Aldrich) for 15 min at 37 °C, then hematoxylin staining. Histological specimens were observed using a microscope (BZ-X710, Keyence). The size of lipid droplets in WAT was quantified using Keyence software (BZ-X-Analyzer, Keyence).

### Quantitative PCR

Total RNA in WAT and liver was extracted using the Lipid RNeasy plus Mini Kit (Qiagen). cDNA was synthesized using an iScriptTMcDNA Synthesis Kit (Bio-Rad). Real-time quantitative PCR was performed using SYBR Green PCR Master Mix (Takara) and primers specific for the target genes of interest.; *CD36*, *Fasn*, *Srebf1*, *TBP*, *Srebp1c*, *Srebp2*, *AdipoQ*, *Leptin*, *F4/80*, *Mcp-1*, *Hmgcr*, *Mvd*, *Hsd17b7*, *Dhcr7*, *StAR*, *StARD3*, *Srebp1a*. *TBP* was used as an internal control. TBP was not affected by Utx deficiency or diet. The results using other internal controls (actin and gapdh) were the same results as TBP. Primer sequences are listed in Supplementary Table 3. Data were collected using a StepOnePlus Real-time PCR system (Applied Biosystems). The PCR reaction was an initial incubation of 95 °C for 30 sec, followed by 40 cycles of PCR (95 °C for 5 sec, 60 °C for 30 sec). The specificity of the detected signals was confirmed by the presence of a dissociation curve containing a single peak and by electrophoresis of PCR products.

### Steroid hormone measurement

Concentrations of E1, E2, A-dion, T, P4, and P5 in frozen mouse adipose tissues were measured using LC-MS/MS at ASKA Pharma Medical Co., Ltd (Kawasaki, Japan) as described previously^53^. Briefly, frozen mouse adipose tissues were homogenized by Ultra-Turrax homogenizer and E1–^13^C_4_ and E2-^13^C_4_ were added as internal standards. Then, E1 and E2 were extracted by methyl *tert*-butyl ether from the mouse adipose suspension. The extract was then applied to Oasis MAX cartridge (Waters, Milford, MA). After repeated washes, the steroids were eluted with methanol/distilled water/pyridine (90:10:1,v/v/v). After evaporation, the residue was then subjected to derivatization. After evaporation, the residue was further reacted with 0.14 mL of derivatized solution [1 mg of 2-methyl-1-hydradino-pyridine in 2.6 mL acetonitorile and 0.2 mL of acetonitorile/trifluoro acetic acid (99:1, v/v)] for 1 h at room temperature. After the reaction sample was evaporated to dryness, the residue was dissolved in 0.5 mL of methanol and diluted with 0.5 ml of distilled water, and then applied to Oasis WCX cartridge that had been successively conditioned with 3 ml of methanol, 3 ml of distilled water. After the cartridge was washed with 1 mL of 0.1 M KH_2_PO_4_ solution, 2 mL of distilled water and 1 mL of 40% acetonitrile solution, respectively, E2-3-tetrafluoropyridine was eluted with 1 mL of methanol. The cartridge was further washed with 2 mL of acetonitorile, 0.5 mL of distilled water and methanol/distilled water/formic acid (65:35:2,v/v/v), derivatized E1 [E1-3-tetrafluoropyridil-17-(1′-methylpyridinium-2′)-hydrazone, E1-TfpyHMP] was eluted with methanol/formic acid (50:1,v/v). After each fraction was evaporated, the derivatized E1 fraction was dissolved in 0.1 mL of acetonitrile/ distilled water (7:3, v/v) and 20 µL of the solution was subjected to an LC-MS/MS. The derivatized E2 fraction was further reacted with 50 μL of mixed solution (80 mg of 2-methyl-6-nitrobenzoic anhydride, 20 mg of 4-dimethylaminopyridine, and 40 mg of fusaric acid in 1 ml of acetonitrile) and 10 μl of TEA for 30 min. at room temperature. After the reaction, the sample was dissolved in 0.5 ml of hexane/acetic acid (50:1, v/v) and the mixture was applied to InertSep SI cartridge, which had been successively conditioned with 3 mL of ethyl acetate and 3 mL of hexane. The cartridge was washed with 1 mL of hexane and 2 ml of ethyl acetate/hexane (3:17, v/v), and then the derivatized E2 (E2-3-tetrafluoropyridil-17-fuzarinoyl ester, E2-TfpyFU) were eluted with 2.5 mL of ethyl acetate /hexane (9:11, v/v). After evaporation, the residue was dissolved in 0.1 mL of acetonitrile/ distilled water (4:1, v/v) and 20 µL of the solution was subjected to LC-MS/MS. An API-5000 triple stage quadrupole mass spectrometer equipped with a positive ESI source and an Agilent1290 Infinity LC system was employed. Capcellcore PFP (2.7 μm, 50 × 2.1 mm i.d.) and Coretecs C18 (1.6 μm, 2.1 × 150 mm) were used at 50 °C. The mobile phase consisting of 0.1% formic acid solution and acetonitrile was used with a gradient elution. The SRM transitions of E2 and E2-^13^C_4_ were *m/z* 583.2/308.1 and 587.2/311.8, respectively. The LLOQ was 0.005 pg/tube.

### RNA sequencing, gene expression profiling, and pathway enrichment analysis

Total RNA was extracted using an RNeasy Lipid Tissue Mini Kit (Qiagen) with an RNase-Free DNase Set (Qiagen). RNA samples had an RNA Integrity Number (RIN) of more than 7.2 confirmed with 2100 Bioanalyzer (Agilent Technologies). Total RNA was converted into a library using SureSelect Strand-Specific RNA library preparation kit (Agilent Technologies). The quality of libraries was checked using 2100 Bioanalyzer and KAPA Library Quantification Kits for NGS. Transcriptome analysis was performed using a next-generation sequencer (HiSeq 2500; Illumina) according to the manufacturer’s instructions. RNA sequence data have been deposited in DDBJ database (accession number: DRA007278). The Illumina’s filter passed sequence tags were mapped onto mouse genomic sequences (UCSC Genome Browser, version mm10) using TopHat2 software. Mapped sequenced tags were assembled using Cufflinks software with upper-quartile-normalization option as previously described^54,55^ and differential expressed genes were analyzed with Cuffdiff command with default setting in Cufflinks. Analyzed data was visualized with CummeRbund package (http://compbio.mit.edu/cummeRbund/) version 2.22.0^56^. Principal component analysis (PCA) was performed using the PCAplot tool in the cummeRbund package. The Heapmap for the genes obtained by Cufflinks analysis was created with Heatplusp R package by the complete and euclidean method. Significant changes (q value < 0.05) in transcript expression between groups and the FPKM (Fragments Per Kilobase of exon per Million mapped reads) of isoforms were calculated and output by Cufflinks^54,56,57^. First, pathway analysis was performed using the Ingenuity Pathway Analysis (IPA) tool (Qiagen) inputting gene sets with significance. Next, Gene Set Enrichment Analysis (GSEA) performed as previously described^58,59^ was used to identify enriched Canonical Pathways analyzing the FPKM of isoforms over 0.3 in all samples.

### Immunoblotting

Whole cell lysates were prepared with CHAPS buffer [40 mM HEPES (pH7.5), 120 mM NaCl, 1 mM EDTA, 0.3% CHAPS, 50 mM NaF, 1.5 mM Na_3_VO_4_, 10 mM glycerophosphate, 10 mM pyrophosphate, 1 mM PMSF] supplemented with protease inhibitor cocktail (Roche Diagnostics, Basel, Switzerland) and phosphatase inhibitor cocktail (Roche Diagnostics). Protein concentration was measured using the BCA assay (Thermo Scientific Pierce). Protein samples were separated by SDS-PAGE and transferred to PVDF membranes (Millipore) using the wet transfer system (Bio-Rad). Membranes were treated with BlockingOne blocking buffer (Nacalai Tesque) for 1 hr and incubated with primary antibody in Can Get Signal (Toyobo) overnight. After 3 washes in TBS-T (Tris buffered saline (pH 7.4) with 0.1% Tween 20), membranes were incubated with secondary antibodies against mouse or rabbit IgG with horseradish peroxidase in Can Get Signal for 1 hr. ECL Prime Western Blotting Detection reagent (Amersham) was used to detect immune complexes. Images were analyzed by Amersham Imager 600 (GE Healthcare Life Science). Primary antibodies recognizing the following proteins were used: Utx, phospho-HSL (Ser563), HSL, ATGL (all from Cell Signaling Technologies); beta-actin (Santa Cruz).

### Statistical analysis

Data are presented as the mean ± SEM. All statistical analyses were performed using unpaired two-tailed Student’s *t*-test or Welch’s correction. A value of *p* < 0.05 was considered significant in all tests. *p < 0.05, **p < 0.005. No statistical methods were used to predetermine sample size. The *in vivo* experiments were not blinded, and the investigators were not blinded to sample allocation during experiments and outcome assessment.

## Supplementary information

Dataset 1

## References

[1] Stein AD, Zybert PA, van de Bor M, Lumey LH (2004). Intrauterine famine exposure and body proportions at birth: the Dutch Hunger Winter. Int J Epidemiol.

[2] Dabelea D (2008). Association of intrauterine exposure to maternal diabetes and obesity with type 2 diabetes in youth: the SEARCH Case-Control Study. Diabetes Care.

[3] Waterland RA, Jirtle RL (2004). Early nutrition, epigenetic changes at transposons and imprinted genes, and enhanced susceptibility to adult chronic diseases. Nutrition.

[4] Tobi EW (2014). DNA methylation signatures link prenatal famine exposure to growth and metabolism. Nat Commun.

[5] Maes HH, Neale MC, Eaves LJ (1997). Genetic and environmental factors in relative body weight and human adiposity. Behav Genet.

[6] Osuga J (2000). Targeted disruption of hormone-sensitive lipase results in male sterility and adipocyte hypertrophy, but not in obesity. Proc Natl Acad Sci USA.

[7] Schreiber R (2015). Hypophagia and metabolic adaptations in mice with defective ATGL-mediated lipolysis cause resistance to HFD-induced obesity. Proc Natl Acad Sci USA.

[8] Rosen ED, Spiegelman BM (2006). Adipocytes as regulators of energy balance and glucose homeostasis. Nature.

[9] Brown MS, Goldstein JL (1997). The SREBP pathway: regulation of cholesterol metabolism by proteolysis of a membrane-bound transcription factor. Cell.

[10] Horton JD (1998). Activation of cholesterol synthesis in preference to fatty acid synthesis in liver and adipose tissue of transgenic mice overproducing sterol regulatory element-binding protein-2. J Clin Invest.

[11] Key TJ (2003). Body mass index, serum sex hormones, and breast cancer risk in postmenopausal women. J Natl Cancer Inst.

[12] Sorensen MB, Rosenfalck AM, Hojgaard L, Ottesen B (2001). Obesity and sarcopenia after menopause are reversed by sex hormone replacement therapy. Obes Res.

[13] Salpeter SR (2006). Meta-analysis: effect of hormone-replacement therapy on components of the metabolic syndrome in postmenopausal women. Diabetes Obes Metab.

[14] Kritz-Silverstein D, Barrett-Connor E (1996). Long-term postmenopausal hormone use, obesity, and fat distribution in older women. JAMA.

[15] Tchernof A (1995). Reduced testosterone and adrenal C19 steroid levels in obese men. Metabolism.

[16] Jones L, Hoban P, Metcalfe P (2001). The use of the linear quadratic model in radiotherapy: a review. Australas Phys Eng Sci Med.

[17] Jones ME (2000). Aromatase-deficient (ArKO) mice have a phenotype of increased adiposity. Proc Natl Acad Sci USA.

[18] Takeda K (2003). Progressive development of insulin resistance phenotype in male mice with complete aromatase (CYP19) deficiency. J Endocrinol.

[19] Jiang W, Wang J, Zhang Y (2013). Histone H3K27me3 demethylases KDM6A and KDM6B modulate definitive endoderm differentiation from human ESCs by regulating WNT signaling pathway. Cell Res.

[20] Seenundun S (2010). UTX mediates demethylation of H3K27me3 at muscle-specific genes during myogenesis. EMBO J.

[21] Miyake N (2013). MLL2 and KDM6A mutations in patients with Kabuki syndrome. Am J Med Genet A.

[22] Lederer D (2012). Deletion of KDM6A, a histone demethylase interacting with MLL2, in three patients with Kabuki syndrome. Am J Hum Genet.

[23] Niikawa N (1988). Kabuki make-up (Niikawa-Kuroki) syndrome: a study of 62 patients. Am J Med Genet.

[24] Philip N (1992). Kabuki make-up (Niikawa-Kuroki) syndrome: a study of 16 non-Japanese cases. Clin Dysmorphol.

[25] Matsumoto N, Niikawa N (2003). Kabuki make-up syndrome: a review. Am J Med Genet C Semin Med Genet.

[26] Devriendt K, Lemli L, Craen M, de Zegher F (1995). Growth hormone deficiency and premature thelarche in a female infant with kabuki makeup syndrome. Horm Res.

[27] Bereket A (2001). Two patients with Kabuki syndrome presenting with endocrine problems. J Pediatr Endocrinol Metab.

[28] Kuroki Y (1987). Precocious puberty in Kabuki makeup syndrome. J Pediatr.

[29] Ota K (2017). The H3K27 demethylase, Utx, regulates adipogenesis in a differentiation stage-dependent manner. PLoS One.

[30] Wang C (2012). UTX regulates mesoderm differentiation of embryonic stem cells independent of H3K27 demethylase activity. Proceedings of the National Academy of Sciences of the United States of America.

[31] He W (2003). Adipose-specific peroxisome proliferator-activated receptor gamma knockout causes insulin resistance in fat and liver but not in muscle. Proc Natl Acad Sci USA.

[32] O’Brien SN, Mantzke KA, Kilgore MW, Price TM (1996). Relationship between adipose stromal-vascular cells and adipocytes in human adipose tissue. Anal Quant Cytol Histol.

[33] Duncan RE, Ahmadian M, Jaworski K, Sarkadi-Nagy E, Sul HS (2007). Regulation of lipolysis in adipocytes. Annu Rev Nutr.

[34] D’Eon TM (2005). Estrogen regulation of adiposity and fuel partitioning. Evidence of genomic and non-genomic regulation of lipogenic and oxidative pathways. J Biol Chem.

[35] Lacasa D, Agli B, Pecquery R, Giudicelli Y (1991). Influence of ovariectomy and regional fat distribution on the membranous transducing system controlling lipolysis in rat fat cells. Endocrinology.

[36] Wohlers LM, Jackson KC, Spangenburg EE (2011). Lipolytic signaling in response to acute exercise is altered in female mice following ovariectomy. J Cell Biochem.

[37] Benoit V, Valette A, Mercier L, Meignen JM, Boyer J (1982). Potentiation of epinephrine-induced lipolysis in fat cells from estrogen-treated rats. Biochem Biophys Res Commun.

[38] Pedersen SB, Borglum JD, Moller-Pedersen T, Richelsen B (1992). Effects of *in vivo* estrogen treatment on adipose tissue metabolism and nuclear estrogen receptor binding in isolated rat adipocytes. Mol Cell Endocrinol.

[39] Pasquier YN, Pecquery R, Giudicelli Y (1988). Increased adenylate cyclase catalytic activity explains how estrogens “*in vivo*” promote lipolytic activity in rat white fat cells. Biochem Biophys Res Commun.

[40] Smith S, Gagne HT, Pitelka DR, Abraham S (1969). The effect of dietary fat on lipogenesis in mammary gland and liver from lactating and virgin mice. Biochem J.

[41] Baverstock PR, Spencer L, Pollard C (1976). Water balance of small lactating rodents–II. Concentration and composition of milk of females on ad libitum and restricted water intakes. Comp Biochem Physiol A Comp Physiol.

[42] Knight CH, Maltz E, Docherty AH (1986). Milk yield and composition in mice: effects of litter size and lactation number. Comp Biochem Physiol A Comp Physiol.

[43] Jeffery E (2014). Characterization of Cre recombinase models for the study of adipose tissue. Adipocyte.

[44] Kang S, Kong X, Rosen ED (2014). Adipocyte-specific transgenic and knockout models. Methods Enzymol.

[45] Berry R, Rodeheffer MS (2013). Characterization of the adipocyte cellular lineage *in vivo*. Nat Cell Biol.

[46] Di Gennaro G, Condoluci C, Casali C, Ciccarelli O, Albertini G (1999). Epilepsy and polymicrogyria in Kabuki make-up (Niikawa-Kuroki) syndrome. Pediatr Neurol.

[47] Franceschini P (1993). Lower lip pits and complete idiopathic precocious puberty in a patient with Kabuki make-up (Niikawa-Kuroki) syndrome. Am J Med Genet.

[48] Ge K (2012). Epigenetic regulation of adipogenesis by histone methylation. Biochim Biophys Acta.

[49] Abel ED (2001). Adipose-selective targeting of the GLUT4 gene impairs insulin action in muscle and liver. Nature.

[50] Kang C (2018). JMJD2B/KDM4B inactivation in adipose tissues accelerates obesity and systemic metabolic abnormalities. Genes Cells.

[51] Copps KD (2010). Irs1 serine 307 promotes insulin sensitivity in mice. Cell Metab.

[52] Mathews ST (2002). Improved insulin sensitivity and resistance to weight gain in mice null for the Ahsg gene. Diabetes.

[53] Ikeda K (2016). Synchronous Multiple Lung Adenocarcinomas: Estrogen Concentration in Peripheral Lung. PLoS One.

[54] Trapnell C (2010). Transcript assembly and quantification by RNA-Seq reveals unannotated transcripts and isoform switching during cell differentiation. Nat Biotechnol.

[55] Roberts A, Trapnell C, Donaghey J, Rinn JL, Pachter L (2011). Improving RNA-Seq expression estimates by correcting for fragment bias. Genome Biol.

[56] Trapnell C (2012). Differential gene and transcript expression analysis of RNA-seq experiments with TopHat and Cufflinks. Nat Protoc.

[57] Trapnell C (2013). Differential analysis of gene regulation at transcript resolution with RNA-seq. Nat Biotechnol.

[58] Subramanian A (2005). Gene set enrichment analysis: a knowledge-based approach for interpreting genome-wide expression profiles. Proc Natl Acad Sci USA.

[59] Mootha VK (2003). PGC-1alpha-responsive genes involved in oxidative phosphorylation are coordinately downregulated in human diabetes. Nat Genet.

