# Supplementary material for: High Fat Diet Triggers a Reduction in Body Fat Mass in Female Mice Deficient for Utx demethylase
Source: Sci Rep. 2019 Jul 11;9:10036. doi: 10.1038/s41598-019-46445-9 (PMC6624269; doi:10.1038/s41598-019-46445-9)
Supplement: Supplementary file 1 — Dataset 1 [file 41598_2019_46445_MOESM1_ESM.pdf]

Supplementary Information:

Supplementary Figures 1-7
Supplementary Tables 1-3

High Fat Diet Triggers a Reduction in Body Fat Mass in Female Mice Deficient for Utx demethylase

Kazushige Ota, Akiyoshi Komuro, Hisayuki Amano, Akinori Kanai, Kai Ge, Takeshi Ueda and Hitoshi Okada

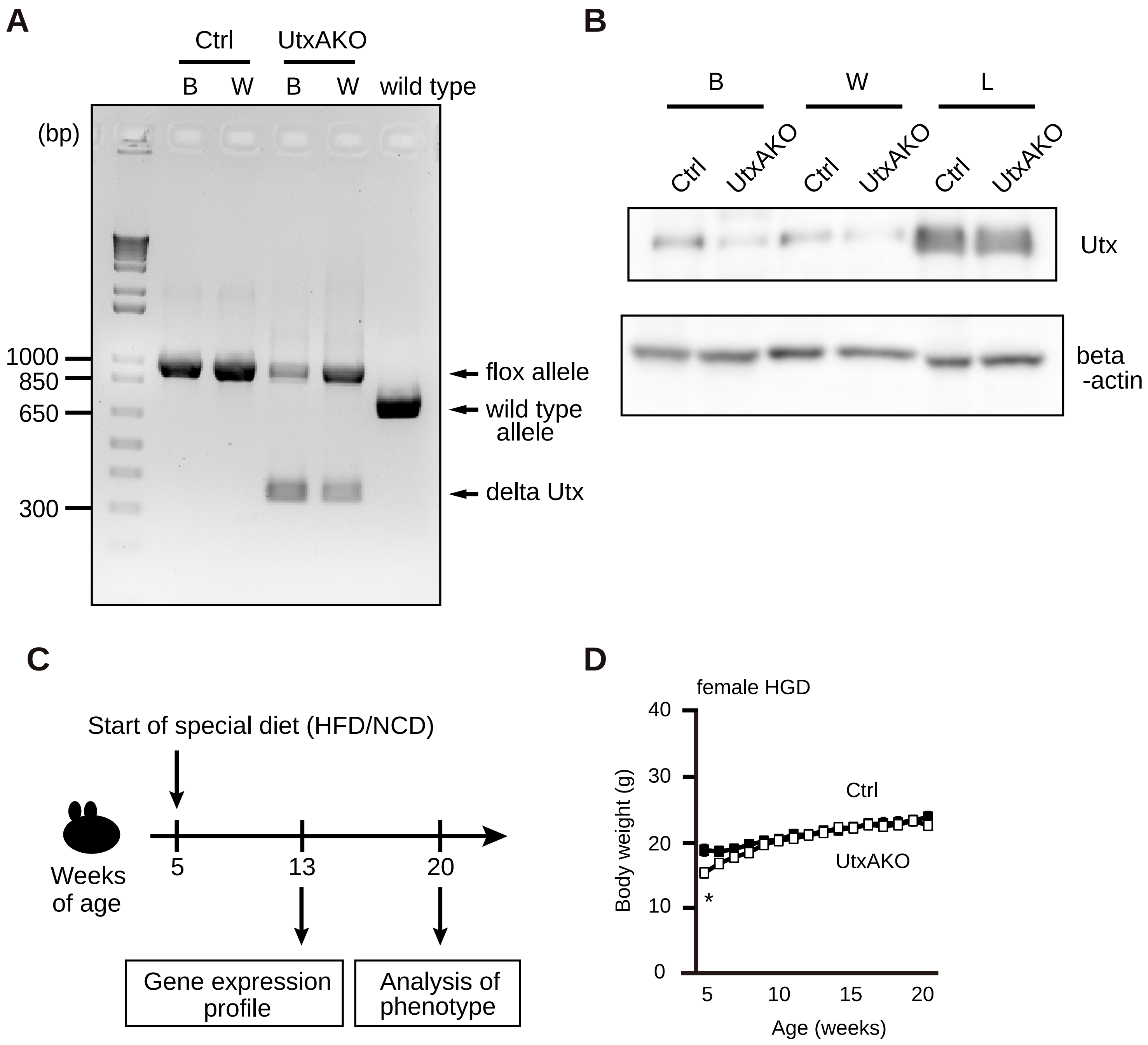

Supplementary Figure 1. Generation and validation of UtxAKO mice. (A) PCR genotyping of brown adipose tissue (BAT; B) and white adipose tissue (WAT; W) in control (Ctrl) and adipocyte-specific Utx-deficient (UtxAKO) female (f) mice fed on NCD. (B) Immunoblot to detect Utx protein in WAT (W), BAT (B), and liver (L) of f-NCD-Ctrl and f-NCD-UtxAKO mice at 8-10 weeks of age. beta-actin, loading control. (C) Schematic diagram of the time course of analyses used to evaluate the phenotypes of UtxAKO mice on NCD or HFD. Cohorts of Ctrl and UtxAKO mice were switched from NCD (or not) to HFD at 5 weeks of age. Gene expression analyses were performed at 13 weeks of age (8 weeks on HFD technically, the NCD mice were 13 weeks on NCD), and gross examinations were performed usually at 20 weeks of age (15 weeks on HFD). (D) Time course of BW gain in female Ctrl and UtxAKO mice fed on a high glucose diet (HGD) (n=5/group).

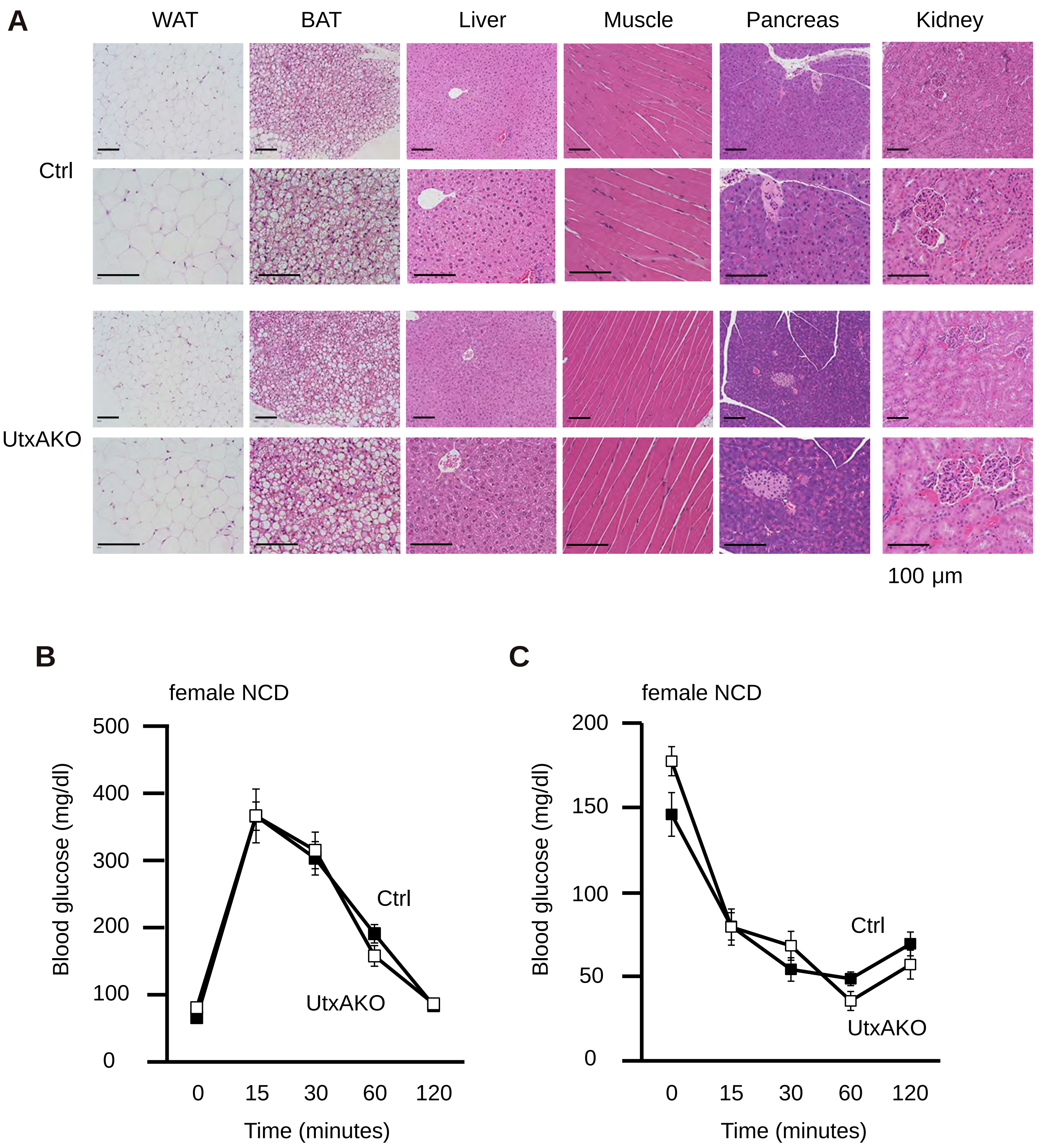

Supplementary Figure 2. UtxAKO mice exhibit comparable phenotypes to Ctrl mice when fed on a normal chow diet (NCD). (A) Histology of the indicated tissues of f-NCD-Ctrl and f-NCD-UtxAKO mice at 8-10 weeks (n=3–5/group). Two magnifications are shown (scale bars, 100 µm). (B) Glucose tolerance test of f-NCD-Ctrl and f-NCD-UtxAKO mice at 8-10 weeks (n=8/group). (C) Insulin tolerance test of f-NCD-Ctrl and f-NCD-UtxAKO mice at 8-10 weeks (n=8/group).

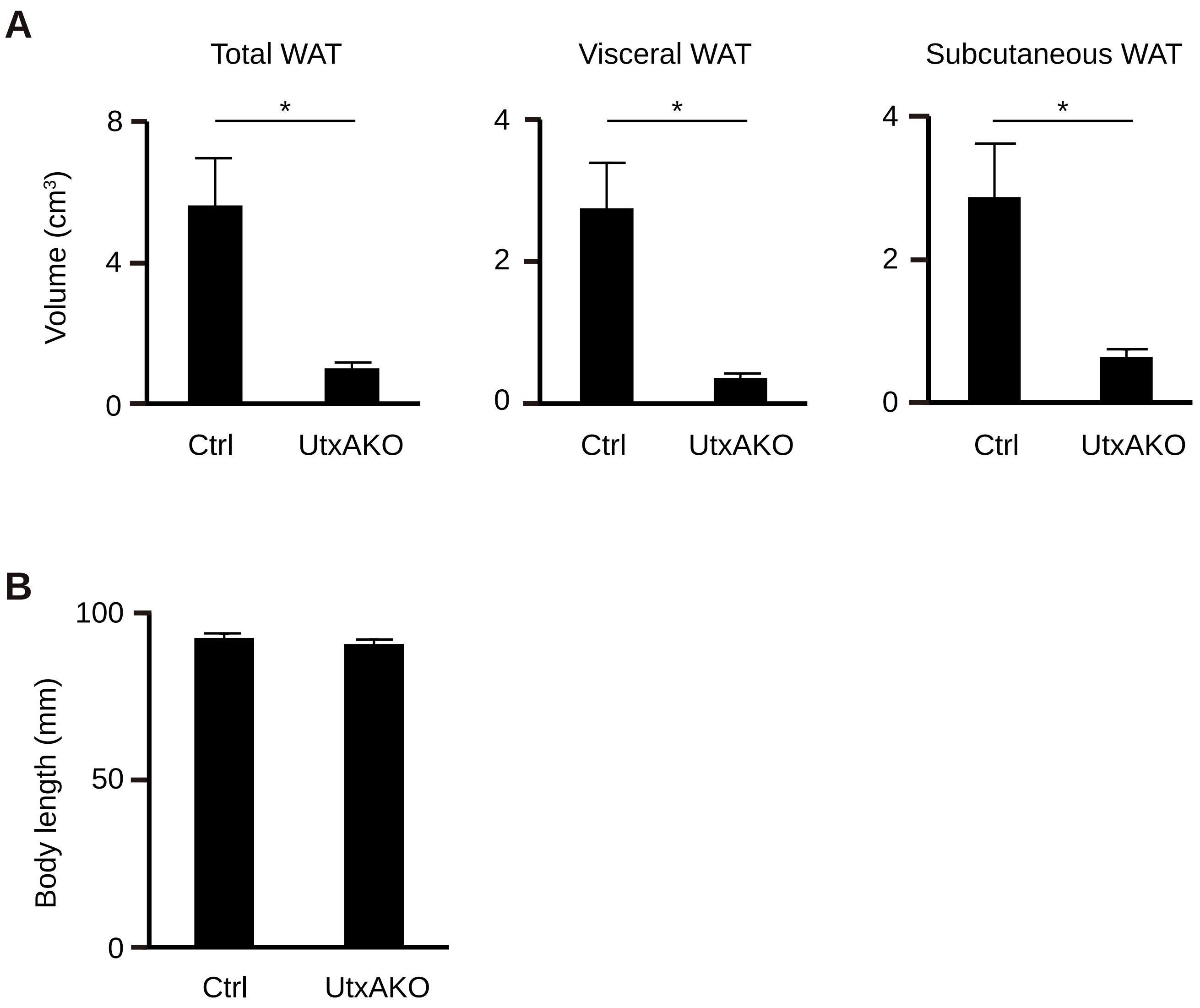

Supplementary Figure 3. Female UtxAKO mice fed on a HFD show reduced volume of adipose tissue. (A) Quantitation of volume of total WAT, visceral WAT and subcutaneous WAT in f-HFD-Ctrl and f-HFD-UtxAKO mice at 20 weeks (n=7/group). (B) Body length of f-HFD-Ctrl and f-HFD-UtxAKO mice at 20 weeks (n=7/group).

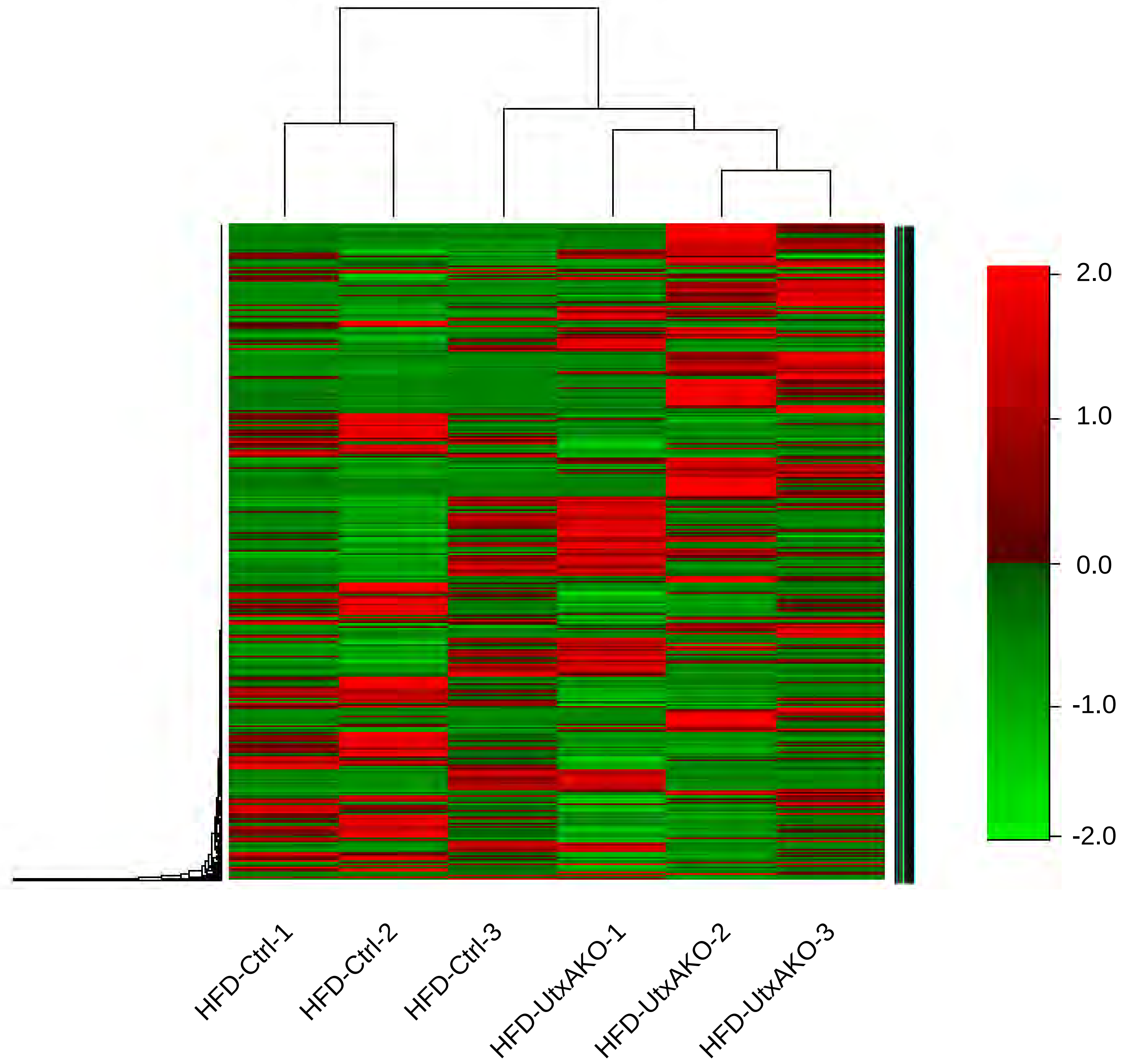

Supplementary Figure 4. Differential expression of transcripts in HFD-fed Ctrl versus UtxAKO visceral adipose tissues. Unsupervised clustering of gene expression patterns in visceral WAT of f-HFD-Ctrl and f-HFD-UtxAKO mice at 13 weeks, as indicated (n=3). Right side: Corresponding heat map of barcode representation. Red and green correspond to high and low expression levels, respectively, compared with the experiment-wide median.

Hormone biosynthetic process

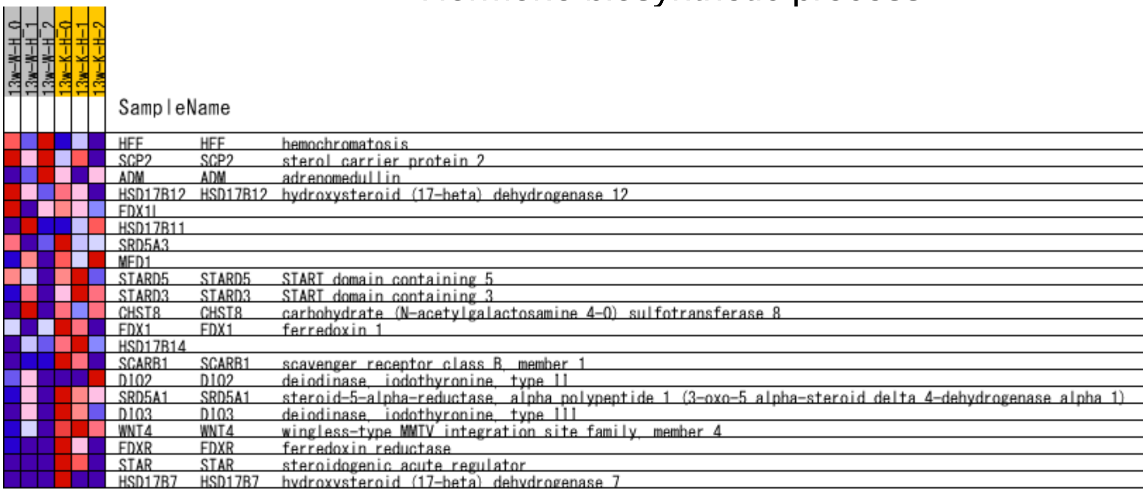

Steroid biosynthetic process

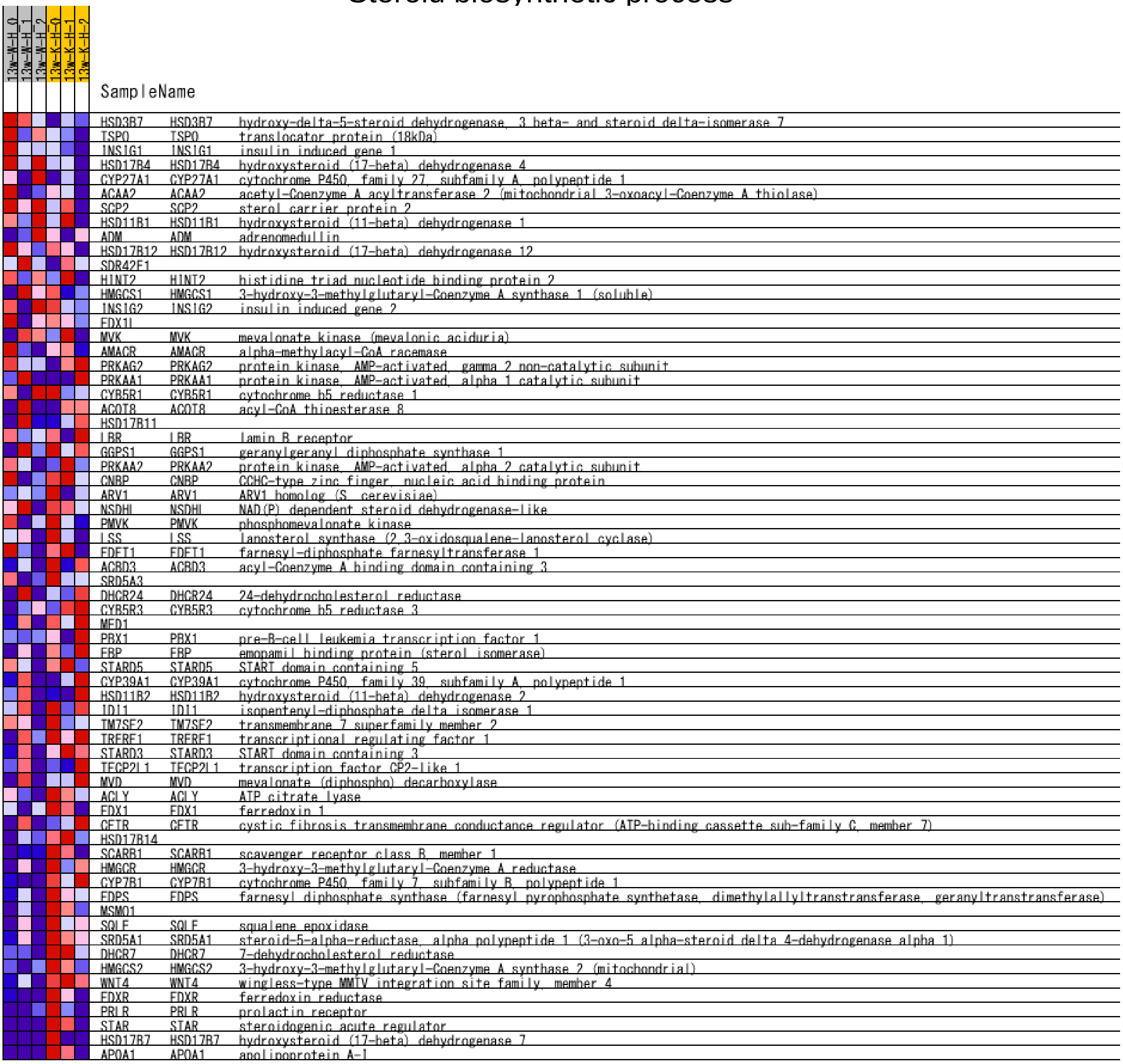

Supplementary Figure 5. Utx deficiency enhances the pathways of cholesterol and estrogen synthesis. The gene lists of representative results from GSEA analysis. The hits of the genes involved in hormone biosynthetic process and steroid biosynthetic process are shown.

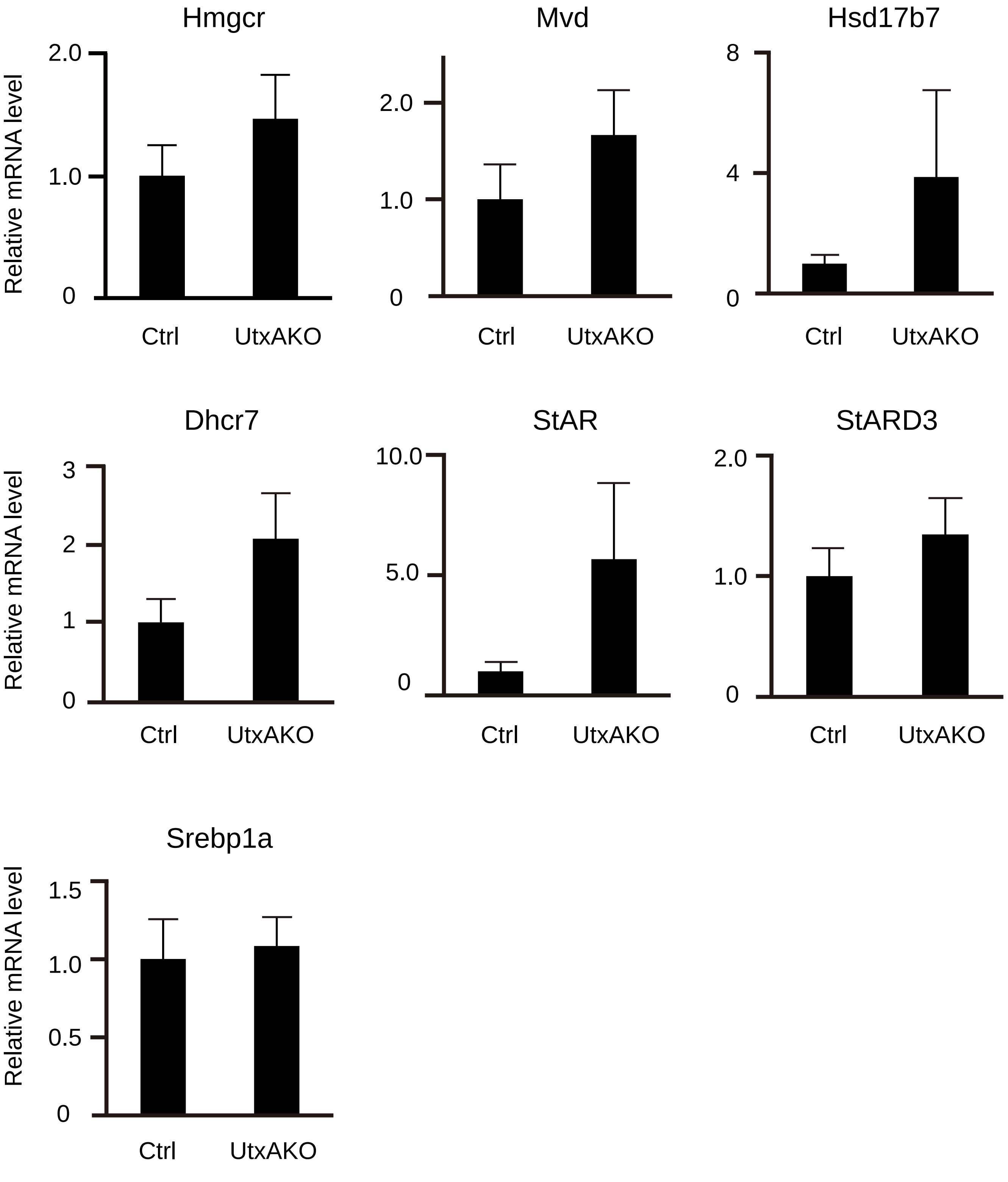

Supplementary Figure 6. Utx regulates a subset of genes responsible for cholesterol synthesis and estrogen metabolism. Relative mRNA levels of the indicated genes in visceral WAT of f-HFD-Ctrl and f-HFD-UtxAKO mice at 13 weeks of age (n=5/group). Results are expressed relative to the mRNA level of TBP (internal control).

Figure 4B

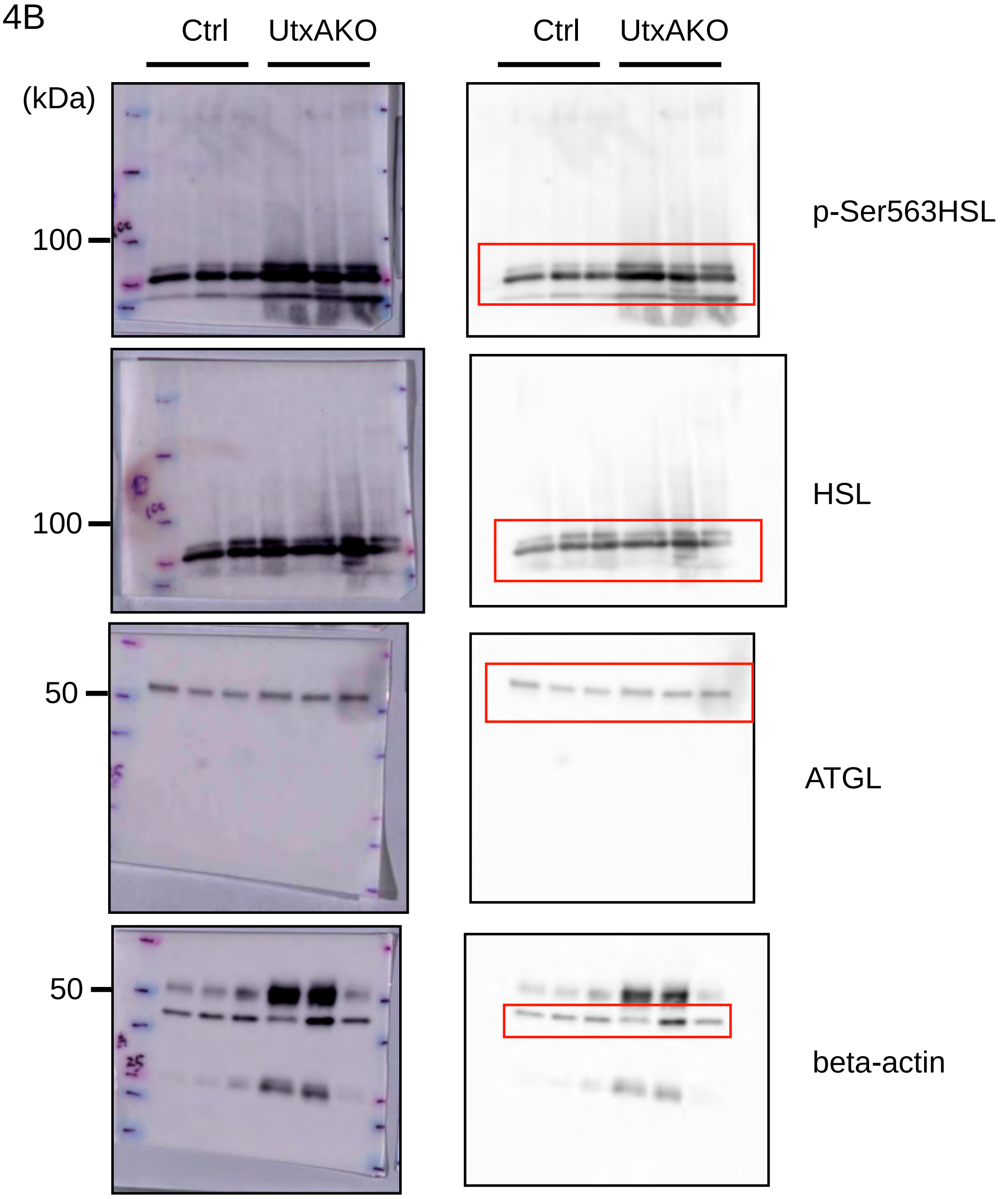

Supplementary Figure 1B

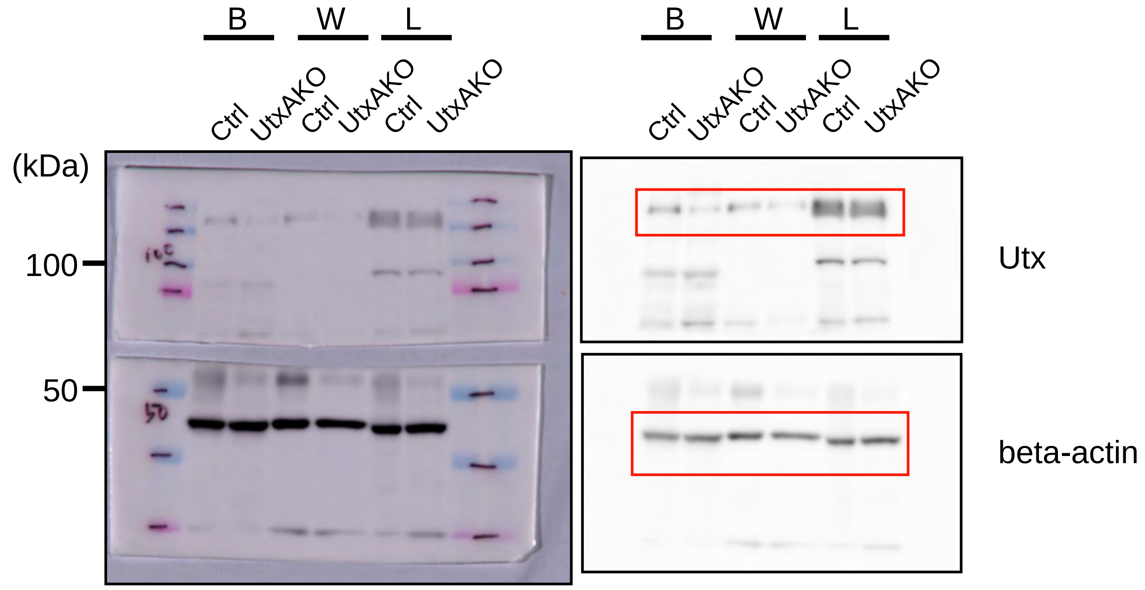

Supplementary Figure 7. Full-length blot images. Full-length blot images related to Figure 4B and Supplementary Figure 1B are shown. The overlaid chemiluminescence images on the paired-visible light images indicate the molecular weight of the bands (left). The cropped regions are indicated (right).

Supplementary Table 1

List of body weights of f-HFD-Ctrl and f-HFD-UtxAKO mice

	Time (week)															
	5	6	7	8	9	10	11	12	13	14	15	16	17	18	19	20
Ctrl 1	20	19.8	20.8	22.2	22.3	24.7	25.3	27.2	27.8	28.9	29.6	30.8	31.1	31.3	33.1	34
Ctrl 2	18.6	19.8	21.3	22.3	23.4	25.3	26.7	27.6	28.4	30.3	31.6	33.5	33.4	35.1	35.9	37.2
Ctrl 3	18.4	18.5	19.9	21.2	22.5	24.3	24.5	25	27.3	27.3	28.2	29	30.5	28.8	29.6	30.5
Ctrl 4	16.4	16.1	17.8	19.5	20.3	21.8	21.6	23.2	23.7	23.9	24.6	24.6	26.5	28.2	29.6	31.3
Ctrl 5	15.4	18.6	19.7	20.5	21.2	22.6	23.4	25.9	26.3	29.1	30.8	32.3	34.1	34	35.7	37.8
Ctrl 6	15	18.2	18.3	19.2	20.1	21	22	22.1	22.1	23	23.7	25.6	25.9	26.4	28	28.3
Ctrl 7	17.3	18.2	20.6	20.6	23.1	22.5	23.5	25	26.9	29.3	30.2	32.3	34.1	35.5	36.4	37.9
Ctrl 8	13.8	16.2	16.6	16.8	17.2	17.3	18.4	19.2	19.7	20	20.3	21.3	21.8	23	24.4	26.1
Ctrl 9	16.2	17.8	19.3	20	21	21.3	22.7	23.1	24.8	25.2	26.2	27.6	28.8	29.4	30.1	31
Ctrl 10	20	19.4	19.6	20.6	21.5	22.5	23.4	23.5	24	26	26.5	27	27	28.1	29.3	30
UtxAKO 1	12.3	16.4	16.4	18.4	18.7	19.8	19.9	20.7	20.7	21.1	22	22.2	22.1	21.6	21.4	22.3
UtxAKO 2	13.2	16.5	18.5	19	20.5	21.1	21.9	22.1	22.8	23.3	23.5	23.8	24.4	24.1	24.9	24.6
UtxAKO 3	17.7	19.7	20	21.4	22.1	22.8	23.4	23.7	24.6	23.4	24.4	24.1	23.6	22.9	22.8	22.2
UtxAKO 4	15.8	18.1	19.4	19.9	21.3	21	20.5	20.6	20.5	20.2	20.6	20.3	20.1	20.9	20	19.9
UtxAKO 5	16	19.4	20.6	21.6	22.6	24.3	25.1	25.6	25.6	23.8	23.6	24.1	23.2	22.5	21.9	21.6
UtxAKO 6	11.8	16.4	18.6	19.1	20.1	21.2	21.9	22.3	22	22.7	23.1	23	23.7	23.3	23.3	23.3
UtxAKO 7	11.1	16	18.1	19.1	19.7	20.4	21.3	21.1	22.1	21.9	22.7	22	22.5	21.6	21.1	22.2
UtxAKO 8	15.4	17.9	18	19.8	19.9	20.6	21.9	22.9	22.4	22.5	23.2	22.4	22.4	22	22.9	21.4
Average																
Ctrl	17.11	18.26	19.39	20.29	21.26	22.33	23.15	24.18	25.1	26.3	27.17	28.4	29.32	29.98	31.21	32.41
UtxAKO	14.1625	17.55	18.7	19.7875	20.6125	21.4	21.9875	22.375	22.5875	22.3625	22.8875	22.7375	22.75	22.3625	22.2875	22.1875
SD																
Ctrl	2.115787	1.305714	1.45331	1.592657	1.809358	2.281836	2.255487	2.512988	2.771281	3.30387	3.59291	3.913509	4.065519	4.007715	3.93572	4.137216
UtxAKO	2.374229	1.445189	1.308216	1.158123	1.30432	1.457003	1.63658	1.694318	1.761037	1.23281	1.156889	1.298282	1.319091	1.044629	1.515103	1.376785
SE																
Ctrl	0.669071	0.412903	0.459577	0.503642	0.572169	0.72158	0.713248	0.794677	0.876356	1.044775	1.136178	1.23756	1.28563	1.267351	1.244584	1.308302
UtxAKO	0.839417	0.510952	0.462524	0.409459	0.461147	0.515128	0.578618	0.599032	0.62262	0.435864	0.409022	0.459012	0.466369	0.369332	0.53567	0.486767

Supplementary Table 2

CE-2

**Nutritional composition**	
Moisture (%)	8.84
Crude protein (%)	25.48
Crude fat (%)	4.61
Crude fiber (%)	5.14
Crude ash (%)	7.01
NFE (%)	48.92
Energy (kcal)	339.1

HFD32

**Nutritional composition**	
Moisture (%)	6.2
Crude protein (%)	25.5
Crude fat (%)	32.0
Crude fiber (%)	2.9
Crude ash (%)	4.0
NFE (%)	29.4
Energy (kcal)	507.6
Fat kcal (%)	56.7

AIN-76

**Nutritional composition**	
Milk casein (%)	20.0
DI-methionine (%)	0.3
Cornstarch (%)	15.0
Granulated sugar (%)	50.0
Cellulose powder (%)	5.0
Corn oil (%)	5.0
Vitamin mix (%)	1.0
Mineral mix (%)	3.5
Choline bitartrate (%)	0.2

Supplementary Table 3

Primers for genotyping

**Name**	**Forward**	**Reverse**
Cre	TAGTTACCCCCAGGCTAAGTG	CTGCCACGACCAAGTGACAG
Utx	AACAAAAACCCAGGCTTTATTCAC	AGTTTCAGGATACCTTTACTATAAG

Primers for real-time RT-PCR

**Name**	**Forward**	**Reverse**
Cd36	TCATGCCAGTCGGAGACATG	TGTCTGTACACAGTGGTGCCTGT
Fasn	TTCCAAGACGAAAATGATGC	AATTGTGGGATCAGGAGAGC
Srebf1	ATCCAGGTCAGCTTGTTTGCGATG	TGGACTACTAGTGTTGGCCTGCTT
TBP	ACCCTTCACCAATGACTCCTATG	TGACTGCAGCAAATCGCTTGG
AdipoQ	TGTTCCTCTTAATCCTGCCCA	CCAACCTGCACAAGTTCCCTT
Leptin	GAGACCCCTGTGTCGGTTC	CTGCGTGTGTGAAATGTCATTG
F4/80	CTTTGGCTATGGGCTTCCAGTC	GCAAGGAGGACAGAGTTTATCGTG
Mcp-1	TTAAAAACCTGGATCGGAACCAA	GCATTAGCTTCAGATTTACGGGT
Srebp1c	GGAGCCATGGATTGCACATT	GAAGTCACTGTCTTGGTTGTTG
Srebp2	CACAATATCATTGAAAAGCGCTAC CGGTCC	TTTTTCTGATTGGCCAGCTTCAGC ACCATG
Hmgcr	AGCTTGCCCGAATTGTATGTG	TCTGTTGTGAACCATGTGACTTC
Mvd	CCGGTCAACATCGCAGTTATC	TTGTGGTCGTTTTTAGCTGGT
Hsd17b7	CCACCTGTGTTTGGCGTGTA	GAGGTTGAATTGTGGATTAGGCA
Dhcr7	AGGCTGGATCTCAAGGACAAT	GCCAGACTAGCATGGCCTG
StAR	AGATGTGGGCAAGGTGTTTC	TGATGACCGTGTCTTTTCCA
StARD3	GTGACTTGGAGCGCAGTTTG	GCCAGTGTTGGTATTTAGCTCG
Srebp1a	GGCCGAGATGTGCGAACT	TTGTTGATGAGCTGGAGCATGT
